# A New Acyl-homoserine Lactone Molecule Generated by *Nitrobacter winogradskyi*

**DOI:** 10.1038/srep22903

**Published:** 2016-03-11

**Authors:** Qiuxuan Shen, Jie Gao, Jun Liu, Shuangjiang Liu, Zijun Liu, Yinghuan Wang, Baoyuan Guo, Xuliang Zhuang, Guoqiang Zhuang

**Affiliations:** 1 CAS Key Laboratory of Environmental Biotechnology, Research Center for Eco-Environmental Sciences, Chinese Academy of Sciences, Beijing 100085, China; 2 State Key Laboratory of Environmental Chemistry and Ecotoxicology, Research Center for Eco-Environmental Sciences, Chinese Academy of Sciences, Beijing 100085, China; 3 State Key Laboratory of Microbial Resources, Institute of Microbiology, Chinese Academy of Sciences, Beijing 100101, China

## Abstract

It is crucial to reveal the regulatory mechanism of nitrification to understand nitrogen conversion in agricultural systems and wastewater treatment. In this study, the *nwiI* gene of *Nitrobacter winogradskyi* was confirmed to be a homoserine lactone synthase by heterologous expression in *Escherichia coli* that synthesized several acyl-homoserine lactone signals with 7 to 11 carbon acyl groups. A novel signal, 7, 8-*trans*-*N*-(decanoyl) homoserine lactone (C10:1-HSL), was identified in both *N. winogradskyi* and the recombined *E. coli*. Furthermore, this novel signal also triggered variances in the nitrification rate and the level of transcripts for the genes involved in the nitrification process. These results indicate that quorum sensing may have a potential role in regulating nitrogen metabolism.

Nitrification is a crucial component of the global nitrogen cycle and has an important role in the conversion of nitrogen in fertilizer in agricultural systems and nitrogen elimination in wastewater treatment^1^. Nitrification is a two-step process. Ammonia-oxidizing bacteria (AOB) and nitrite-oxidizing bacteria (NOB) are considered to be the most important contributors to nitrification and always appear to work together to convert ammonia into nitrate^2^,^3^. However, recent studies indicated that ammonia-oxidizing archaea (AOA) are also widely distributed in various types of ecosystems and play important roles in the global nitrogen biogeochemical cycle^4^,^5^. *Nitrobacter winogradskyi* is a key member of the NOB family that oxidizes nitrite into nitrate to obtain energy for growth^6^,^7^. Additionally, as a facultative chemolithoautotroph, *N. winogradskyi* can also utilize organic compounds as their sole carbon and energy sources^1^. During the microbial nitrification process, AOB, NOB and other bacteria could form a biofilm to efficiently oxidize ammonia and nitrite^8^,^9^. Because bacteria use small molecules to assess the density and identity of nearby organisms, some signal transduction mechanisms that act via bioactive molecules may be the strategy to regulate their metabolism and physiological behavior to adapt to various conditions. Biofilm formation is controlled by a cell density-dependent quorum sensing (QS) mechanism in many Gram-negative bacteria^10^. Recent studies have indicated that long-chain QS signals could increase the anoxic ammonium oxidation rate in the oxygen-limited autotrophic nitrification/denitrification (OLAND) process^11^, and NO_3_^−^ reduction and N_2_ production were decreased after C4-HSL was added to QS mutants of *Pseudomonas aeruginosa* PAO1, which indicated that the denitrification activity was controlled by *rhl*-mediated QS in *P. aeruginosa*^12^. Increasing evidence indicates that QS is a global regulation system in some *Proteobacteria* strains^13^. These studies have raised questions of whether the QS system could have major consequences in regulating nitrogen metabolism.

QS is a cell-cell communication mechanism that employs autoinducers to regulate bioluminescence, biofilm formation, swarming, plasmid transfer and exoenzyme secretion^14^,^15^,^16^. QS bacteria can sense and respond to cell density variations to regulate gene expression. Acyl-homoserine lactone (AHL) molecules are primarily biosynthesized by the LuxI protein family, and then QS-relative genes can be activated in response to the interaction between the AHL molecules and the cognate LuxR regulator protein^17^,^18^,^19^. The environmental concentration of AHLs is the key factor that triggers the response^20^. Three AHL synthase families have been characterized: the LuxI family, the AinS family and the HdtS family^21^. Based on the homology of known QS system genes, the genome sequence of *N. winogradskyi* revealed that the nwi_0626 and nwi_0627 genes were predicted to encode a pair of LuxI and LuxR homologs that are responsible for the synthesis of AHLs as a QS signal and a responsive transcription factor, respectively^1^,^22^. A previous study isolated two AHLs from *N. winogradskyi* in response to cell density^23^. However, the authors were unable to clarify the structure of the presumed unsaturated acyl-HSL, and the function of the postulated signal synthase (nwi_0626) currently remains unclear. Although the AHL-producing activity was confirmed in *N. winogradskyi*, the AHL synthase, LuxI/R QS system and the postulated QS in *N. winogradskyi* have not been proven. We found that different AHL types were produced by this *Nitrobacter* in a recent preliminary study and examined the DNA sequence of this bacterium to identify *luxI* homologs in this species. The polypeptide encoded by nwi_0626 (NwiI) clusters with homologs of the LuxI subfamily, which could enzymatically synthesize a QS signal with a unique structure (Supplementary Figure S1 A). To investigate the mechanism by which this *Nitrobacter* produces different acyl-HSL quorum-sensing signals and the possibility that the QS system may serve to regulate the physiological functions of *N. winogradskyi*, we sought to identify the differences in AHL synthesis in various growth conditions and the products of the AHL synthase activity.

Here, we describe the potential AHL synthase (NwiI) of *N. winogradskyi*, which belongs to the LuxI family. When *nwiI* was introduced into *Escherichia coli*, various types of AHLs were identified in the extracts of the recombinant strain. Additionally, we discovered that *N. winogradskyi* produced different types of AHL signals under different culture conditions, including an acyl-homoserine lactone (HSL) with an unsaturated C10 acyl side chain that clearly demonstrates a specific configuration. The molecule also exhibits a different position for the double bond compared with the previously reported structure^23^. The addition of the C10:1-HSL extract to the culture medium revealed that it influenced the process of nitrification. These results demonstrate that the nitrite-oxidizing bacteria *N. winogradskyi* has an acyl homoserine lactone-based QS system that is responsible for the biosynthesis of various AHLs with a series of saturated and unsaturated acyl chains, and the predominant autoinducer C10:1-HSL has the capacity to regulate the nitrite oxidation process.

## Results

### Identification of the *nwiI* gene responsible for AHL production

**Figure 1:**
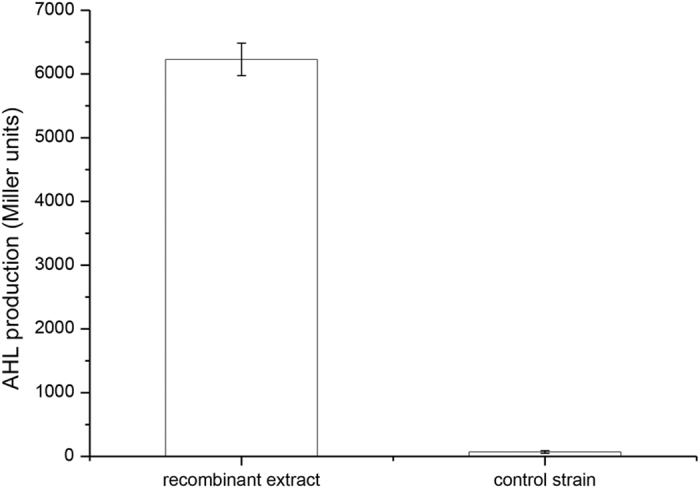
AHL production (Miller units) from the recombinant extract and control *E. coli* extract in *A. tumefaciens* KYC55 (pJZ372) (pJZ384) (pJZ410) using the liquid assay. The values are the mean of three independent biological replicates. The error bars indicate mean ± standard deviation of three biological replicates determinations.

**Figure 2:**
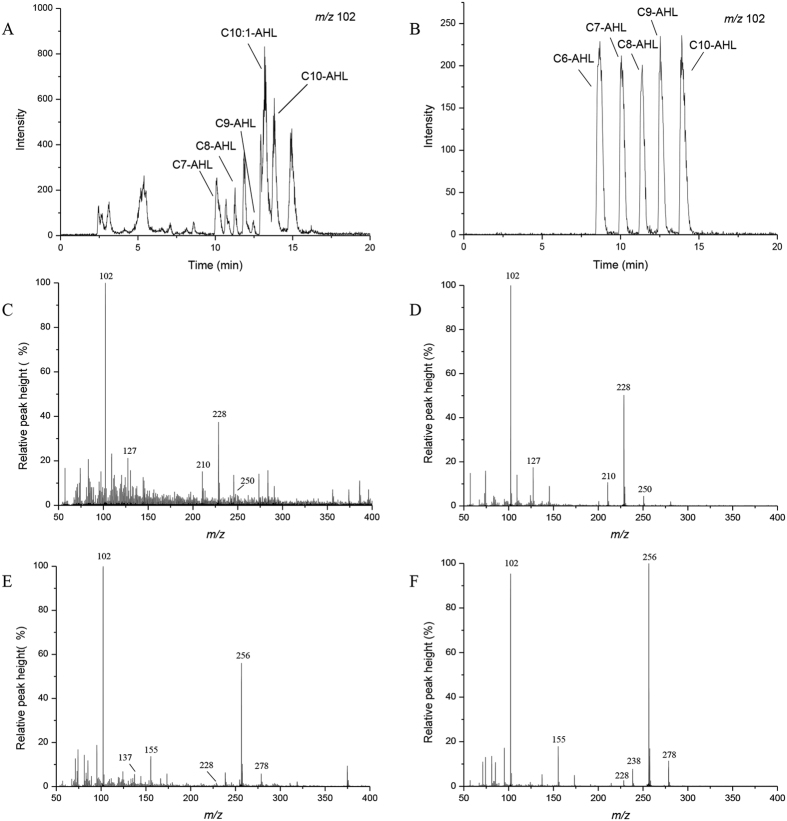
LC-MS chromatograms of the AHL molecules in *E. coli* containing pGEX-nwiI and the AHL standards. (**A**) Chromatogram of the lactone moiety at *m*/*z* 102 from an extract of the recombinant *E. coli* strain. (**B**) Selected ion (*m*/*z* 102) chromatograms for the C6-HSL, C7-HSL, C8-HSL, C9-HSL and C10-HSL standards. (**C,E**) The mass spectra of the extracts from recombinant *E. coli* containing pGEX-nwiI reveal molecular ions [M+H] of *m*/*z* 228 and 256. The comparable fragmentation products are labeled with their respective *m*/*z* values. (**D,F**) The fragmentation patterns of the extract of the recombinant strain were consistent with those of the C8-HSL (**D**) and C10-HSL (**F**) standards.

**Figure 3:**
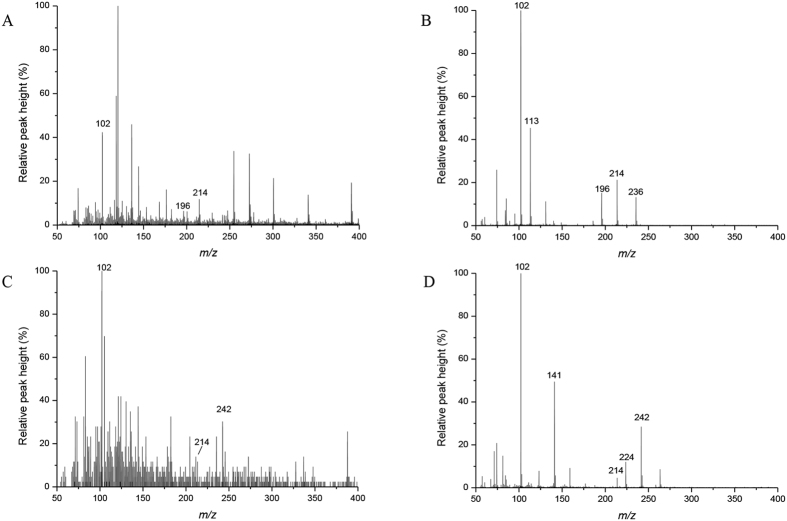
LC-MS chromatograms of C7-HSL and C9-HSL in *E. coli* containing pGEX-nwiI. (**A,C**) The mass spectra of extracts from recombinant *E. coli* containing pGEX-nwiI reveal molecular ions [M+H] of *m*/*z* 214 and 242. The comparable fragmentation products are labeled with their respective *m*/*z* values. (**B,D**) The fragmentation patterns of the extract from the recombinant strain were consistent with those of the C7-HSL (**B**) and C9-HSL (**D**) standards.

To investigate the potential AHL synthase from *N. winogradskyi*, it is necessary to compare, clone, and heterologously express the homologs from *N. winogradskyi*. The sequence for the putative QS signal synthase NwiI from *N. winogradskyi* was conserved among the group of AHL synthase proteins. According to the analysis of the 16S and 23S rRNA gene sequences, the alphaproteobacterium *N. winogradskyi* is closely related to phototrophic bacterium *Rhodopseudomonas palustris* CGA009 and the dinitrogen-fixing legume symbiont *Bradyrhizobiaceae japonicum* USDA110^1^. The phylogenetic tree of the LuxI and HdtS family members revealed that the NwiI polypeptide sequence was closely related to the AHL synthetases BraI, BjaI and RapI from *Bradyrhizobium* and *Rhodopseudomonas* (Supplementary Figure S2). The open reading frame (ORF) (nwi_0626) encoded a protein that was 39% and 25% similar to the BjaI protein from *B. japonicum* USDA110 and the LasI protein from *Pseudomonas aeruginosa* PAO1, respectively (Supplementary Figure S1 A). Moreover, the open reading frame (ORF) (nwi_0627) encoded a protein that was 33 and 31% similar to the BjaR protein from *B. japonicum* USDA110 and LasR protein from *Pseudomonas aeruginosa* PAO1, respectively (Supplementary Figure S1 B). Based on the structural similarities between NwiI and the autoinducer synthases employed by other bacteria, NwiI contained the same conserved amino acid residues that were important for the active sites as the proteins (BjaI and LasI) with known QS functions^24^,^25^. Next, *nwiI* was expressed in *E. coli*, which does not produce AHLs. The extracts of LB medium alone were also performed by the LC-MS, and no AHLs were detected (Supplementary Figure S3 A). The *nwiI* gene was cloned into the pGEX-4T-1 plasmid and transformed into the *E. coli* BL21 (DE3) strain. The AHL bioassay strain *A. tumefaciens* KYC55 uses the T7 expression system to strongly overexpress the regulator TraR. When AHL signals are present, TraR can activate a traI-*lacZ* reporter fusion protein, resulting in the production of β-galactosidase^26^. The signals obtained from the recombinant sample turned the biosensor supernatant solution yellow, and no activity was observed in the control *E. coli* strain carrying pGEX-4T-1 without the *nwiI* gene. The levels of *lacZ* induction by AHLs were then reported in Miller units (Fig. 1). This finding suggested that *nwiI* from *N. winogradskyi* encoded an AHL synthase when overexpressed in the heterologous host *E. coli* BL21(DE3). A positive result was obtained in the bioassays, and high-performance liquid chromatography-mass spectrometry (HPLC-MS) was used to identify the acyl chains of the AHLs. The retention time and mass spectra of the AHLs in the recombinant extract were compared with AHL standards. Here, we confirmed that four different acyl chains of AHLs were present in the recombinant extract. Two compounds with an even number of *N*-linked acyl chains were clearly identified with molecular ions [M+H]^+^ of *m*/*z* 228 and 256 that correspond to C8-HSL and C10-HSL, respectively (Fig. 2). These two AHLs were previously identified in a number of Gram-negative bacteria^27^,^28^. In addition, we also identified two AHLs with an odd number of carbons in the acyl chain with molecular ions [M+H]^+^ of *m*/*z* 214 and 242 that correspond to C7-HSL and C9-HSL, respectively (Fig. 3). The AHLs with an odd number of *N*-linked acyl chains used propionyl-CoA and malonyl-CoA as the respective chain starter and extender units via a pathway that produces odd chain fatty acids^29^. However, the odd-numbered AHLs were not as prevalent as the even-numbered AHLs.

### Structural analysis of a new AHL molecule with an unsaturated C10 acyl side chain

**Figure 4:**
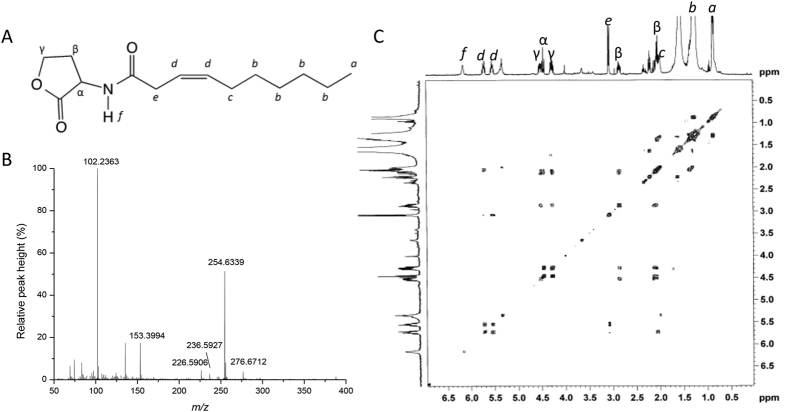
Predicted structure (**A**), LC-MS (**B**), and proton NMR and COSY spectrum (**C**) purified from the extracts of the recombinant *E. coli* strain containing the *nwiI* gene. The protons are indicated by italicized letters and correspond to the following peaks in the ^1^H-NMR (CDCl_3_, 400 MHz) spectrum: δ_H_ 0.88 (3H, t, a,), 1.29 (8H, m, b), 2.01 (2H, m, c), 2.11 (1H, m, β), 2.86 (1H, m, β), 3.08 (2H, d, e), 4.28 (1H, m, γ), 4.47 (1H, t, α), 4.53 (1H, m, γ), 5.54 (1H, m, d), 5.73 (1H, m, d), and 6.17 (1H, m, f).

As described above, there were eight autoinducer molecules produced by *E. coli* containing pGEX-nwiI (Fig. 2A). Only four of the molecules were identified as standard AHLs (C7-HSL, C8-HSL, C9-HSL and C10-HSL). We purified the other four putative AHLs using preparative HPLC. The analysis of the highest mass spectrum peak revealed pseudomolecular ion peaks for [M + H]^+^ and [M + Na]^+^ at *m*/*z* 254 and *m*/*z* 276, respectively, with major fragmentation ions at *m*/*z* 102 and *m*/*z* 153 and minor fragmentation ions at *m*/*z* 226 ([M + H-CO]^+^) and 236 ([M + H-H_2_O]^+^) (Fig. 4B). This mass could not be attributed to any known AHL, and this putative AHL molecule might possess an unsaturated acyl chain compared with the mass spectrum of C10-HSL. The proton nuclear magnetic resonance (^1^H NMR) spectrum of the putative purified AHL was similar to the other AHLs, and all of the protons could be unambiguously assigned (Supplementary Figure S4)^30^,^31^. According to the ^13^C nuclear magnetic resonance (^13^C NMR) spectrum (Supplementary Figure S5), we predicted that this molecule contained a 10-carbon acyl group with one unsaturated carbon-carbon (*trans* configuration) bond. As determined by correlation spectroscopy (COSY), the double bond in the acyl chain was located between carbons 7 and 8 (Fig. 4). The HPLC-MS results revealed that the *m*/*z* of [M + H]^+^ was 254.6339. This value corresponded to a chemical composition of C_14_H_23_NO_3_, which was consistent with the structure presented in Fig. 4.

In addition to the five identified AHL molecules (C7-HSL, C8-HSL, C9-HSL, C10-HSL and C10:1-HSL), three probable AHL molecules were difficult to identify due to the lack of standards. In addition, the extremely low concentrations and interference from other similar compounds that were likely aromatics influenced the NMR spectrum (data not shown). Generally, using MS detection, the AHLs decompose into two main fragments, a [M + H]^+^ ion from neutral loss of homoserine lactone and the *m*/*z* 102 ion from protonation of the homoserine lactone. Furthermore, two other protonations of the HSLs lead to the [M + H-H_2_O]^+^ and [M + H-CO]^+^ ions^32^. Although the remaining components in the solution have not yet been purified, it could be hypothesized that the recombinant *E. coli* strain containing *nwiI* produced C8:1-HSL, C9:1-HSL and C11:1-HSL, according to the liquid chromatography-mass spectrometry (LC-MS) chromatograms (Supplementary Figure S6).

### Characterization of the AHLs produced by *N. winogradskyi* under different growth conditions

**Figure 5:**
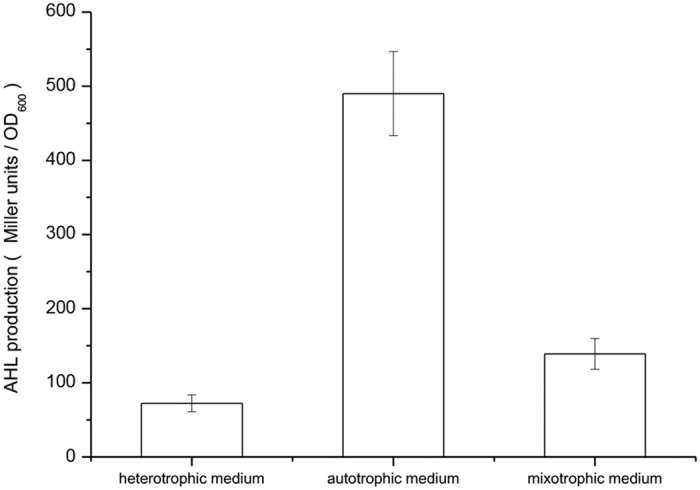
AHL production (Miller units/OD_600_) in *N. winogradskyi* in heterotrophic medium, autotrophic medium and mixotrophic medium by *A. tumefaciens* KYC55 (pJZ372) (pJZ384) (pJZ410) using the liquid assay. The values are the mean of three independent biological replicates. The error bars indicate mean ± standard deviation of three biological replicates determinations.

**Figure 6:**
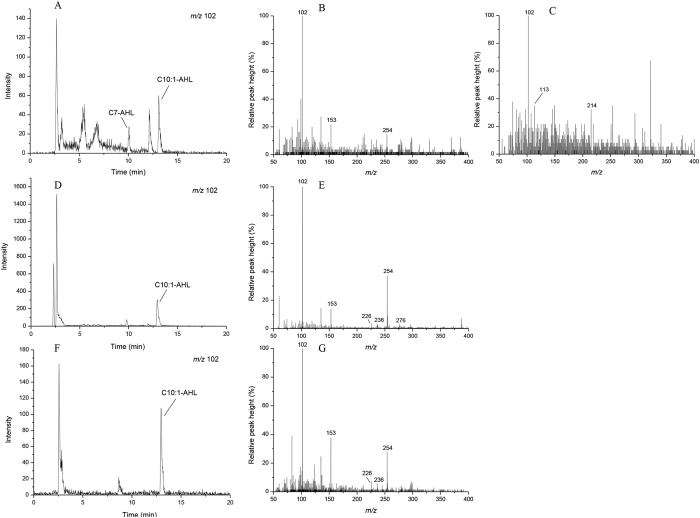
LC-MS chromatograms of the AHLs molecules in *N. winogradskyi* under different culture conditions. (**A**) Chromatogram of the lactone moiety at *m*/*z* 102 from the *N. winogradskyi* mixotrophic culture extract. (**B**,**C**) The mass spectra of mixotrophic culture extracts reveal molecular ions [M + H] of *m*/*z* 214 and 254. (**D**) Chromatogram of the lactone moiety at *m*/*z* 102 from the *N. winogradskyi* autotrophic culture extract. (**E**) The mass spectra of the autotrophic culture extracts reveal molecular ions [M + H] of *m*/*z* 254. (**F**) Chromatogram of the lactone moiety at *m*/*z* 102 from the *N. winogradskyi* heterotrophic culture extract. (**G**) The mass spectra of the heterotrophic culture extracts reveal molecular ions [M + H] of *m*/*z* 254.

As a facultative chemolithoautotrophic bacterium, *N. winogradskyi* can grow in heterotrophic, mixotrophic and autotrophic culture conditions. Thus, we set out to examine these NOB AHLs in different culture conditions using the bioassay strain *Agrobacterium tumefaciens* KYC55. Positive results were obtained in the bioassay experiments using all three culture mediums. As shown in Fig. 5, in autotrophic medium, the AHL levels were obviously increased compared with the other culture conditions. The amount of acyl-HSL in the autotrophic substrate significantly accumulated and exhibited a five-fold increase in Miller units/OD_600_ compared with the heterotrophic culture. In addition, we also used LC-MS to confirm the AHLs that were present in the *N. winogradskyi* extract. Here, we confirmed that AHLs with two different acyl chains, C7-HSL and C10:1-HSL, were present in the extract of the cells cultured in the mixotrophic medium, whereas only C10:1-HSL was detected in the extracts of the cells cultured in the heterotrophic and autotrophic media (Fig. 6). The extract of culture medium alone were also performed by LC-MS, and no AHLs were detected (Supplementary Figure S3 B).

### 7,8-*trans*-*N*-(decanoyl) homoserine lactone affects the nitrification process

To further characterize the impact of AHLs on the nitrification process in *N. winogradskyi*, 2 μM C10:1-HSL that was purified from the recombinant *E. coli* extracts was added to the *N. winogradskyi* culture medium. The purity of the C10:1-HSL generated by preparative HPLC was confirmed by LC-MS (Supplementary Figure S7). During the logarithmic phase of *N. winogradskyi* growth, the nitrite oxidation rate followed Michaelis-Menten kinetics^33^. Michaelis-Menten kinetics are characterised by the hypothesis that the enzyme and substrate participate in equilibrium with the enzyme-substrate complex^34^. Although the experiments were not performed with purified enzymes, the term *K*_m_ was used to determine nitrite oxidation kinetics, because the kinetics in short-term activity assays over a few hours, where growth can be neglected^33^. After the addition of C10:1-HSL, the half-saturation constant for nitrite oxidation (*K*_m_) was 138.06 ± 27.04 μM. The value for the control group was 143.63 ± 47.25 μM. Although the nitrite affinity in C10:1-HSL addition group was slightly higher than control, no significant differences in the *K*_m_ values were noted (*p* ≥ 0.05). However, the half-lives, t_1/2_, of nitrite were 5.19 h and 4.81 h in the control and C10:1-HSL-treated groups, respectively (Supplementary Table 1). The result indicated that the utilization of nitrite was slightly increased after the addition of C10:1-HSL.

**Figure 7:**
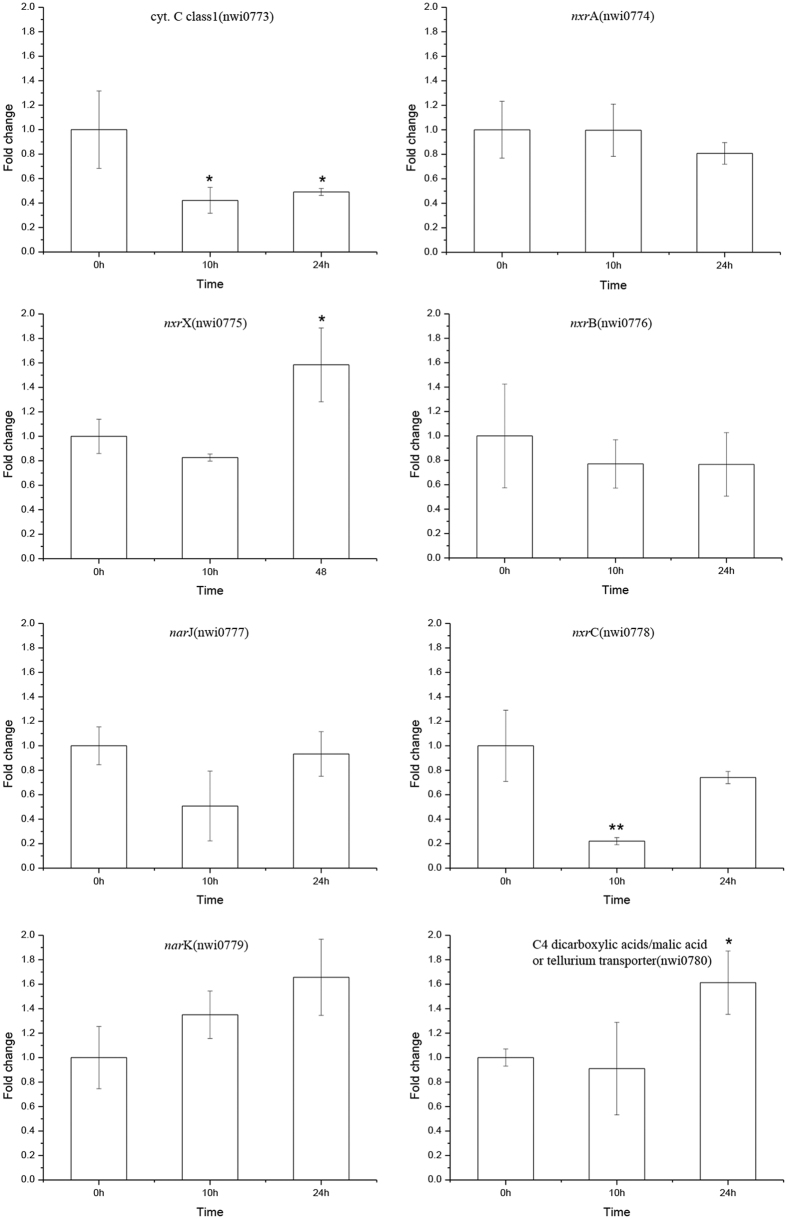
Changes in gene expression after the addition of exogenous AHLs during the culture process. The fold changes in gene expression were measured by qPCR. The stars indicate significant changes in gene expression compared with the samples at 0 h (*p ≤ 0.05, **p ≤ 0.01). Statistical analyses of the ΔCt values were analyzed by SPSS using the two-sample t-test for the means. Results were considered significant when p ≤ 0.05.

The expression of genes in the nitrite oxidoreductase (NXR) cluster, which contains a molybdopterin that converts nitrite to nitrate, was also investigated. NXR is a heterodimer that contains an α subunit (NxrA) and a β subunit (NxrB) and is highly similar to the membrane-bound dissimilatory nitrate reductase (Nar) of *E. coli* and many other bacteria^35^. Two copies of *nxr*A and *nxr*B were present in the *N. winogradskyi* genome, but only one central gene cluster (nwi_0773 to nwi_0780) encodes the accessory proteins of the NXR complex^1^. The nwi_0773 gene is predicted to encode a *c*-type cytochrome that is likely a part of the electron transport system that couples the oxidation and reduction of nitrite and is located upstream of *nxrA*^1^. The *nxr*X (nwi_0775) gene is a homolog of *nor*X, which encodes a peptidyl-prolyl *cis-trans* isomerase to aid in the folding of NXR^1^. Moreover, two genes, *nar*J (nwi_0777) and *nxr*C (nwi_0778), which are downstream of *nxrB*, are predicted to encode homologs of NarJ and NxrC, respectively. NarJ is a chaperone required for insertion of the molybdenum cofactor in nitrate reductase A. And NxrC is a periplasmic cytochrome *c* that serves as the electron acceptor and donor^36^. In addition, a NarK-like nitrate/nitrite transporter (nwi_0779) and transporter for C4 dicarboxylic acids/malic acid or tellurium (nwi_0780) are also conserved in *N. winogradskyi*^1^. The oxidation of nitrite by NXR is reversible, and NXR can also catalyze the reduction of nitrate to nitrite, which is considered to be part of the denitrification pathway in *N. winogradskyi*^37^. Both strains that were treated with autoinducer and blank strains were evaluated for their ability to activate or inactivate the transcription of the NXR cluster genes in *N. winogradskyi.* The quantitative PCR results indicated that the expression levels of *nxrA* (nwi_0774) and *nxrB* (nwi_0776) were not obviously altered in the strain that was incubated with C10:1-HSL (Fig. 7). However, the expression levels of *nxrX* (nwi_0775), *narK* (nwi_0779) and a gene encoding for C4 dicarboxylate transporter (nwi_0780) were increased 24 h after C10:1-HSL was added compared with the samples without the novel signal (Fig. 7). Although *nxrA* and *nxrB* were not activated, transcriptional inhibition of *c*-type cytochrome (nwi_0773) (*p* value: 0.039), *narJ* (nwi_0777) and *nxrC* (nwi_0778) (*p* value: 0.0099) were observed after a 10 h incubation with 2 μM C10:1-HSL (Fig. 7).

## Discussion

To identify the signal types that were produced by the AHLs synthase in *N. winogradskyi*, we introduced *nwiI* into *E. coli*, which resulted in the synthesis of a series of AHLs with chain lengths ranging from C7 to C11. Five of the AHLs were identified as C7-AHL, C8-AHL, C9-AHL, C10-AHL and C10:1-AHL. However, three probable AHL molecules remain to be confirmed due to the lack of standards and the extremely low concentration of the samples. Despite the fact that the remaining components of the solution have not been purified, it could be hypothesized that the recombinant *E. coli* strain containing *nwiI* produced C8:1-AHL, C9:1-AHL and C11:1-AHL according to the LC-MS chromatograms. It is unexpected that one QS signal synthase gene was responsible for the biosynthesis of various AHLs with a series of saturated and unsaturated acyl chains ranging from 7 to 11 carbons in length. According to the MS, NMR and COSY analyses, a new AHL molecule with an unsaturated C10 acyl side chain was discovered. Although C10:1-HSL signaling molecules were analyzed in several reports, the physicochemical characterizations were often incomplete, and a definite structure was merely implied^38^,^39^,^40^. Mellbye *et al.*^23^ presumed that a monounsaturated acyl-HSL (C10:1-HSL) with the C=C bond at the end of the acyl chain was present in *N. winogradskyi* using UPLC-IDA-MS; however, the results were insufficient to clarify the isomeric form of the C=C bond. Our results clearly indicated that *N. winogradskyi* produced a novel type of acyl-HSL, 7,8-*trans*-*N*-(decanoyl) homoserine lactone, which differed from the C10:1-HSL structure, as mentioned above.

As a facultative chemolithoautotrophic bacterium, *N. winogradskyi* can grow in heterotrophic, mixotrophic and autotrophic culture conditions. Thus, we examined these NOB AHLs in different culture conditions. It is interesting that the AHL signals produced by *N. winogradskyi* were inconsistent with the recombinant *E. coli* strain. Schaefer *et al.*^25^ reported that the LuxI homolog of *Rhodobacter capsulatus* synthesized a different type of AHLs in *E. coli* instead of the native AHL signals. *E. coli* containing acyl-HSL synthase synthesized C14-HSL but did not produce the native C16-HSL of *R. capsulatus*. Our study obtained similar results, as many more AHLs were produced by the recombinant *E. coli* compared with *N. winogradskyi*. The biosynthesis of AHLs showed that *S*-adenosylmethionine (SAM) and acyl carrier protein (ACP) charged with the appropriate fatty acid as sources of the homoserine lactone and acyl chain, respectively^41^. The variety of the *N*-acyl chains represents a broad range of modifications that are generated in fatty acid biosynthesis. The LuxI homologue often shows a preference for one specific fatty acyl-ACP derivative leading to the formation of one major AHL product, but closely related AHLs with similar chain lengths frequently occur in the same bacterial species^41^. However, there was lack of mechanism to illuminate different production range of AHLs in heterologous expression strain. The main AHL product of one AHL synthase reflects its preferred substrate, and the metabolically versatile property of *N. winogradsky* probably resulted in these difference. Moreover, in the detection of the AHLs in the original *N. winogradskyi* strain, the amount of AHLs in the autotrophic medium was obviously increased compared with the other culture conditions. The C7-HSL and C10:1-HSL signals were confirmed in the mixotrophic medium extract, whereas only C10:1-HSL was detected in the heterotrophic and autotrophic medium extracts. A recent study has demonstrated that different signals are utilized when the cell densities were high or low, particularly when multiple signals were integrated^40^. The growth-dependent QS study in *P. aeruginosa* demonstrated that the signaling pathway performs differently when the cell densities were different. The integration of multiple signals with different chemical half-lives allowed *P. aeruginosa* to decode its physical and social environment with high resolution^40^. Additionally, it is worth noting that environmental stimuli, particularly the nature of the carbon sources, have a powerful influence on the type of autoinducer molecule produced^42^. *N. winogradskyi* was able to utilize nitrite, carbon dioxide and organic compounds in the absence of nitrate to support growth^1^. This metabolically versatile property likely resulted in the release of different AHLs from *N. winogradskyi* in response to various survival conditions and its own demands.

One interesting phenotype we measured was that the maximum accumulation of AHLs occurred in autotrophic cultures with the highest nitrite concentration (Fig. 5), suggesting a potential role for QS in preparing the organism for higher cell densities by controlling nitrogen metabolism in *N. winogradskyi*. From the results of the growth condition assays in *N. winogradskyi*, C10:1-HSL appeared to be a necessary autoinducer, as it was detected in all growth conditions (Fig. 6). To further characterize the impact of the AHLs on the nitrification process in *N. winogradskyi*, 2 μM purified C10:1-HSL was added to the *N. winogradskyi* culture medium. NxrA/B are the key catalytic subunits for nitrite oxidation in *N. winogradskyi*, as the expression levels of the *nxr*A/B did not respond to AHL. Transcriptional inhibition of *c*-type cytochrome (nwi_0773) and *nxrC* (nwi_0778) were observed in the presence of C10:1-HSL, indicated a reduced NXR activity. However, The *K*_m_ results indicated that the affinities for the substrate nitrite were slightly increased by additional C10:1-HSL in *N. winogradskyi*; thus, other factors must regulate the increased nitrite oxidation activity. The results of Nowka *et al.*^33^ indicated the *Nitrobacter* affinities for nitrite substrate was related to the transporters which shuttled nitrite across the cytoplasmic membrane. The increased expression level of NarK-like nitrate/nitrite transporter (nwi_0779) after the addition of C10:1-HSL may lead to the results described above. Both the active uptake of nitrite or efflux of nitrate would be important for maintaining NXR activity in *N. winogradskyi*; therefore, the effect of adding exogenous C10:1-HSL to the culture medium appeared to activate the nitrification process. Interestingly, combined with the results of the different culture media on the AHL concentrations, these results suggest that the QS system was strongly induced in the nutrient-rich environment and was much more sensitive to nitrite. It is reasonable that the nitrite-rich culture could in fact trigger the production of a sufficient amount autoinducer to reach an effective concentration, and the increasing AHL concentration has a profound effect on accelerating nitrite absorption, which subsequently underlie these results.

In summary, a novel C10:1-HSL molecule, 7,8-*trans*-*N*-(decanoyl) homoserine lactone was generated by the *N. winogradskyi* autoinducer synthase, as confirmed by the MS, NMR and COSY analyses. We measured the effect of adding exogenous C10:1-HSL to the *N. winogradskyi* culture medium, resulting in slightly variance of the nitrification process. Moreover, we cannot exclude the possibility that the different types of AHLs produced by *Nitrobacter* provide an opportunity to study the combinatorial information processing that is regulated by the QS system. The discovery of the ability of *Nitrobacter* to synthesize AHLs opens up additional avenues of research to demonstrate the regulatory role of QS in the global nitrogen cycle. It remains to be investigated whether the QS effects confer a broad advantage to *N. winogradskyi* compared with other environmental nitrifying bacteria.

## Methods

### Bacterial strains and growth media

*N. winogradskyi* (ATCC 25391) was grown in heterotrophic nitrobacter medium 756 (http://www.dsmz.de/microorganisms/medium/pdf/DSMZ_Medium756.pdf), mixotrophic nitrobacter medium 756a (http://www.dsmz.de/microorganisms/medium/pdf/DSMZ_Medium756a.pdf) and autotrophic nitrobacter medium with 60 mM NaNO_2_ minimal salts as previously described^43^. All cultures were checked for contamination by spread plating on nutrient agar (incubated at 26 °C). If no growth on agar occurred after 5 days the cultures were assumed to be axenic. Only data from axenic cultures were used. And the purity of the nitrifying bacteria was confirmed by microscopical examination. *E. coli* BL21(DE3) and its derivatives were grown at 37 °C and 200 rpm in Luria-Bertani (LB) broth or on LB agar containing ampicillin (100 μg/ml), as required. *A. tumefaciens* KYC55 (pJZ372) (pJZ384) (pJZ410) was used as the AHL bioassay strain^26^. The strain was cultivated at 28 °C and 200 rpm in *A. tumefaciens* (AT) medium^44^ containing 100 μg/ml spectinomycin, 100 μg/ml gentamicin, and 5 μg/ml tetracycline. The AT medium contains AT salts with 0.5% glucose^45^.

### Bioinformatic analyses

The GenBank database was searched using the BLAST program (http://blast.ncbi.nlm.nih.gov) to identify the genes encoding AHL synthases. The sequences were aligned and compared using the CLUSTAL W Server (http://www.ch.embnet.org/software/ClustalW.html).

### DNA manipulations

*N. winogradskyi* DNA was prepared from cells grown in mixotrophic nitrobacter medium at 26 °C using a Genomic DNA Purification Kit (Thermo Fisher Scientific, Waltham, USA), according to the manufacturer’s instructions. The DNA concentrations were determined using a Nanodrop spectrophotometer (Nanodrop Technologies, Rockland, DE). DNA isolated from *N. winogradskyi* was used as the template to amplify the *nwiI* gene using the following primers: 5′- CAGGATCCATGATTCACATCGTAACGGC-3′ (*nwiI*-forward) and 5′- CCGCTCGAGCTAGGCACGAAGCCGC-3′ (*nwiI*-reverse). The *nwiI*-forward primer included a BamHI restriction site, and the *nwiI*-reverse primer included an XhoI restriction site (underlined). The PCR conditions were 2 min at 95 °C followed by 30 cycles of 30 s at 95 °C, 30 s at 50 °C, and 1 min at 72 °C with a final step of 10 min at 72 °C. The amplified *nwiI* gene was cloned into pGEX-4T-1 (GE), according to the manufacturer’s instructions. The recombinant pGEX-4T-1 plasmid containing *nwiI* was termed pGEX-nwiI. pGEX-nwiI was transformed into *E. coli* BL21(DE3), and the transformant was grown on LB medium containing ampicillin (100 μg/ml) at 37 °C. The detailed characteristics of the bacterial strains and plasmids used in this study are presented in Supplementary Table S2.

### AHLs bioassays

AHL bioactivity was determined by measuring the β-galactosidase bioactivity of the ultrasensitive AHL biosensor strain *A. tumefaciens* KYC55 using a previously described method^26^. The cultures of recombinant *E. coli* and *N. winogradskyi* were centrifuged at 5,000 × *g* for 5 min to remove the cells. A 0.22-μm syringe filter was used to filter the assayed supernatants. The cell-free culture supernatants were stored at −20 °C until further examination. *A. tumefaciens* KYC55 (5 × 10^7^) were inoculated into 2 ml of *A. tumefaciens* culture medium containing 200 μl of the supernatant of the recombinant *E. coli*^45^. The supernatant of an *E. coli* strain carrying pGEX-4T-1 without the *nwiI* gene was used as a negative control. For each sample, the absorbance at OD_600_ was recorded after approximately 10 h at 28 °C. Then, 200 μl of the supernatant from the AT culture medium was combined with 0.8 ml of Z buffer (60 mM Na_2_HPO_4_, 40 mM NaH_2_PO_4_, 10 mM KCl, 1 mM MgSO_4_, and 50 mM 2-mercaptoethanol, pH 7.0) in 2-ml microcentrifuge tubes. Then, 2 drops of a 0.05% SDS solution were added, and 3 drops of chloroform were added. The samples were vigorously vortexed for 10 s. Then, 0.1 ml of 4 mg/ml ortho-nitrophenyl-β-D-galactopyranoside (ONPG) was added, and the solutions were placed in a 30 °C water bath for 10 min. The reactions were stopped by the addition of 0.6 ml of 1 M Na_2_CO_3_. The cell debris were centrifuged at 16,000 × *g* for 3 min at room temperature, and the OD_420_ of the supernatant was measured. The β-galactosidase units were calculated as follows: Miller units = (1000 × OD_420_)/(OD_600_ × 10 × 0.2), where the OD_420_ was read from the supernatant of the reaction mixture. AHL production in *N. winogradskyi* in the heterotrophic, autotrophic and mixotrophic medium was calculated by Miller units per OD_600_ of culture.

### Extraction, purification and detection of AHLs

The supernatants of the recombinant *E. coli* strain and *N. winogradskyi* were extracted two times with an equal volume of acidified ethyl acetate (EtAc) containing 0.2% glacial acetic acid and finally dried by a rotary evaporator. The extracts and AHL standards were reconstituted in HPLC-grade acetonitrile. LB and 756 medium alone were also extracted and detected using the same methods. The AHLs’ profiles were confirmed with an HPLC-MS system (Waters; USA) using a C18 reverse-phase column (5 μm by 250 mm by 4.6 mm) (Zorbax Eclipse XDB-C18; Agilent) coupled with positive-ion electrospray ionization (ESI) mass spectrometry^32^ and eluted with a linear gradient of acetonitrile in water (10–70%) at a flow rate of 1 ml/min from 0 to 10 min and a linear gradient of acetonitrile in water (70–10%) at a flow rate of 1 ml/min from 10 to 20 min. The retention times and spectral properties of the ESI spectra (*m*/*z* range, 50 to 400) containing a fragment product at *m*/*z* 102 were compared with the corresponding synthetic AHL standards^46^ (Sigma-Aldrich, USA). These extracts were applied to a C18 reverse-phase preparative HPLC column and eluted in 50% acetonitrile at a flow rate of 15 ml/min over a 2-h period by monitoring the spectra at 210 nm. The eluant was collected, and the active fractions were analyzed by LC-MS, ^1^H NMR, ^13^C NMR and COSY. ^1^H NMR and ^13^C NMR was recorded in CDCl_3_ with a Bruker 400 MHz (100 MHz for ^13^C) NMR spectrometer (Bruker Corporation, Germany).

### Calculation of the oxidation kinetics and data analysis

Samples were prepared from cells grown in mixotrophic nitrobacter medium at 26 °C to measure the nitrite concentration. The sample times were 0, 2, 4, 6, 8, 12 and 24 h after treatment with or without 2 μM C10:1-HSL. LC-MS was used to detect the purity of the C10:1-HSL generated by preparative HPLC. The optical densities of the samples were analyzed for the presence of NO^2−^ at 520 nm using sulfanilic acid and α-naphthylamine as reagents^47^. Nitrite oxidation is an enzymatic reaction that happens in the cytoplasm of the cell in *Nitrobacter*, so the characteristics of the nitrite oxidation kinetics were obtained by fitting the data to the Michaelis-Menten kinetics. The Michaelis–Menten constant was calculated using Lineweaver-Burk plots of the double reciprocal of the Michaelis-Menten equation: *V* = (*V*_max_∙[*S*])/(*K*_m_ + [*S*]). Here, *V* is activity, *V*_max_ is the maximum specific activity (μmol/ml/h), *K*_m_ is the half-saturation constant for nitrite oxidation (μM), and [*S*] is the nitrite concentration (μM). The pharmacokinetic parameters were calculated using the decrease in the nitrite concentration. The decrease in the nitrite concentration appeared to follow a pseudo-first-order kinetic reaction. The nitrite concentrations (C) and the sampling time (t) were expressed as follows: lnC = −kt + b. The half-life t_1/2_ of nitrite was calculated as follows: t_1/2_ = 0.693/k.

### RNA extraction and quantitative PCR (qPCR)

The total RNA was prepared from cells grown in mixotrophic nitrobacter medium supplemented with or without 2 μM C10:1-HSL at 26 °C at 0, 10 and 24 h using an RNeasy mini kit (Qiagen, Germantown, MD), according to the manufacturer’s recommendations. The RNA concentrations were determined using a Nanodrop spectrophotometer (Nanodrop Technologies, Rockland, DE). The reverse transcription reaction was performed using a PrimeScript^TM^ RT Master Mix (TaKaRa, Biotechnology, China). Real-time PCR was performed on a CFX96^TM^ Real-Time PCR system (Bio-Rad Laboratories, USA) with SYBR Premix Ex Taq^TM^ (TaKaRa Biotechnology, China). Each reaction was performed in a total reaction mixture of 25 μl containing 12.5 μl of SYBR Premix Ex Taq^TM^, 10 μM of each primer and 1 ng of DNA. The relevant primers are listed in Supplementary Table S3. The fold changes were calculated using the double ΔCT method (i.e., using the equation 2^−ΔΔCt^). The experiments were performed in triplicate. The ΔCt values were statistically analyzed with SPSS software (SPSS Inc., Chicago, USA) using the two-sample *t*-test to determine the differences between the mean values. The results were considered significant when *p* ≤ 0.05.

## Additional Information

**Accession codes:** Sequence data from this study have been deposited in GenBank under accession number KR703654.

**How to cite this article**: Shen, Q. *et al.* A New Acyl-homoserine Lactone Molecule Generated by *Nitrobacter winogradskyi*. *Sci. Rep.* **6**, 22903; doi: 10.1038/srep22903 (2016).

## Supplementary Material

Supplementary Information
