# Supplementary material for: A New Acyl-homoserine Lactone Molecule Generated by Nitrobacter winogradskyi
Source: Sci Rep. 2016 Mar 11;6:22903. doi: 10.1038/srep22903 (PMC4786786; doi:10.1038/srep22903)
Supplement: Supplementary Information [file srep22903-s1.pdf]

A New Acyl-homoserine Lactone Molecule Generated by *Nitrobacter winogradskyi*

Qiuxuan Shen[1]*, Jie Gao[1]*, Jun Liu[2], Shuangjiang Liu[3], Zijun Liu[1], Yinghuan Wang[1], Baoyuan Guo[1], Xuliang Zhuang[1#] & Guoqiang Zhuang[1#]

[1] CAS Key Laboratory of Environmental Biotechnology, Research Center for Eco-Environmental Sciences, Chinese Academy of Sciences, Beijing 100085, China

[2] State Key Laboratory of Environmental Chemistry and Ecotoxicology, Research Center for Eco-Environmental Sciences, Chinese Academy of Sciences, Beijing 100085, China

[3] State Key Laboratory of Microbial Resources, Institute of Microbiology, Chinese Academy of Sciences, Beijing 100101, China

* These authors contributed equally to this work.

# Correspondence and requests for materials should be addressed to X.Z. (email: xlzhuang@rcees.ac.cn) or G.Z. (email: gqzhuang@rcees.ac.cn)

Supplementary Table S1. Pharmacokinetic parameters of the decrease in the nitrite concentration decrease in the control and C10:1-HSL groups

Pharmacokinetic parameters	Control group	C10:1-HSL group
First-order kinetic equation	y=-0.1336x+5.1022	y=-0.1442x+5.1077
R^2	0.95705	0.97785
k	0.1336	0.1442
$t_{1/2}$	5.19	4.81

Supplementary Table S2. Bacterial strains and plasmids used in this study

Strain or plasmid	Description	Reference
Strains		
Nitrobacter winogradskyi ATCC 25391	Type strain	ATCC
Escherichia coli BL21(DE3)	F^- *omp*T *hsd*S_B (r_B^- m_B^-) *dcm gal* λ (DE3) Cm^r	Promega
Agrobacterium tumefaciens KYC55(pJZ372) (pJZ384) (pJZ410)	AHL biosensor strain, Tc^r, Spe^r, Gm^r	[1]
Plasmids		
pGEX-4T-1	*E. coli* cloning and expression vector	GE
pGEX-nwiI	*nwiI* of *N. winogradskyi* expressed from pGEX-4T-1 *tac* promoter	This study

Supplementary Table S3. Primers used in the quantitative PCR

Locus tag	Gene name/description	Primers
nwi0773	cyt. C class 1	5’ TAAGGGTGATGCTGAGAACG (F)
		5’ CTTCATGGTCGGAGTGGG (R)
nwi0774	*nxr*A	5’ CACTCCGCACTTTAATCGTA (F)
		5’ GCGCCAGATGAACCACAT (R)
nwi0775	*nxr*X	5’ TCAACTACGACGTACCCG (F)
		5’ TTACCGCCCGAAGATGCT (R)
nwi0776	*nxr*B	5’ CGGTTCGCAGGTTTACGC (F)
		5’ CCGTCCTCACCACGCTTGTA (R)
nwi0777	*nar*J	5’ CGTGATCTGCGAAAGCAA (F)
		5’ CGTAAAGCATCTAGCACCTGA (R)
nwi0778	*nxr*C	5’ GACTCCCACGGCACTTTA (F)
		5’ CCTGTAAGCCAGCGAAAC (R)
nwi0779	*nar*k	5’ CAGCATTCCACTCGGTATTT (F)
		5’ CCCTGCTTAGACTTCGGATA (R)
nwi0780	C4 dicarboxylic	5’ CACGTCGTGGCGACTTAT (F)
	acids/malic acid or	5’ CATGATCGTCAGGTTAGCG (R)
	tellurium transporter	

A

```
NwiI        MIHIVTAENIGSYQYEMEQAYRLRHNVFVDEMGWDDLRKPDGREIDQFDDGRALHMLYIE
BjaI        MIHAISAVNRHLYEDVLEQHFRLRHDIFVEERHWETLRRPDGREWDSYDDEDTVYLLALE
LasI        -MIVQIGRREEFDKKLLGEMHKLRAQVFKERKGWDVS-VIDEMEIDGYDALSPYYMLIQE
             :    . .     :  : : .:** ::* :.   *:      *  *:* :*    . ::*  *

NwiI        ED---RVLGYQRMLPSMRPHLLTEVLPHLCEG-DFPVGPHIWEWTRYCVTRQHRDRGRIL
BjaI        GR---RVVGGHRLYPTTKPSMMSEVFPHLAAVRGCPSDPLIWEWSRYFVVRDRRD-----
LasI        DTPEAQVFGCWRILDTTGPYMLKNTFPELLHGKEAPCSPHIWELSRFAINSGQKGSLG-F
                 :*.*  *:  :  * ::.:.:*.*      * .* *** :*: :    ::.

NwiI        SPVGNLLLSAIVEWGLASGVQKIIIEMNPLWLLRLVQLNFRVTPLGLPQQIGKDSIIAVT
BjaI        GALNLQLMAAVQEFCLDQGIAQVSAIMETWWLPRFHEAGFVVTPLGLPALVENAWTMAAT
LasI        SDCTLEAMRALARYSLQNDIQTLVTVTTVGVEKMMIRAGLDVSRFGPHLKIGIERAVALR
            .      : *: .: * ..:  :            : . .: *: :*     :     :*

NwiI        AAFDRRTLKKLQEVRGNIVPAIVAQPEADQRLRA-------------
BjaI        VDIRRQTLDVLHDRIGMPSIVQQDGPRLDAVARANLCGLAAAQRKSA
LasI        IELNAKTQIALYGGVLVEQRLAVS-----------------------
              :  :*   *
```

B

```
LasR        -------MALVDGFLELERSSGKLEWSAILQKMASDLGFSKILFGLLPKD---SQDYENA
NwiR        -MSRRTLDDTLTFIARVDKASTPAEIADAVVDVARPLGFSHVLAGIIPIPGMTAEQQISN
BjaR1       MSAVDYGREALDFIEGLGVYRKVPDAMNALEAAFGRFGFETIIVTGLPNP---DQRFAQM
                       :  :  :       :    :      :**. ::   :*      :   .

LasR        FIVGNYPAAWREHYDRAGYARVDPTVSHCTQSVLPIFW---EPSIYQTRKQHEFFEEASA
NwiR        VVLHRWPKAWSERYFTKGYLFDDPTIQRVNTSTEPFLWSELEPTYRNTPAPTRVMGEARE
BjaR1       VLAKRWPAGWFNLYTQNNYDRRDPVVRLCRQSVNPFEWSEAPYDAELEPSAAEVMNRAGD
            .:  .:* .* : *   .*   **.:      *.  *: *               ..: .*

LasR        AGLVYGLTMPLHGARGELGALSLSVEAENRAEANRFIESVLPTLWMLKDYALQSGAGLAF
NwiR        FNLGCGFTVPMITLNGQTAGFSLASERAEVPSILR---------GQLQLIAMYAFARALG
BjaR1       FRMSRGFIVPIHGLTGYEAAVSLGGVHLDLNPRSK-------PALHLMAMYGFDHIRRLL
              :  *: :*:    *  ...**.    :     :            *

LasR        EHPVSKPVVLTSREKEVLQWCAIGKTSWEISVICNCSEANVNFHMGNIRRKFGVTSRRVA
NwiR        QKYKPAPVNLTPREMDILQWMAEGKSDWEISVILKVSEHLVDKIARQLRAKLNATNRTQT
BjaR1       EPTPYPSTRLTPREREVISWASQGKSAWEIGEILHITQRTAEEHLATAARKLGAVNRTHA
            :     .. **.** :::.* : **: ***. * : ::  .:        *:....*  :

LasR        AIMAVNLGLITL
NwiR        VAVALRHNLIR-
BjaR1       VALAIRHKIINP
            . :*:.  :*
```

Supplementary Figure S1. Sequence alignment of NwiI and NwiR. The amino acid sequences of NwiI/R were compared to BjaI/R and LsaI/R, respectively. Identical and similar amino acid residues in the three proteins are indicated by asterisks and colons, respectively, and nine conserved residues in the LuxI/R family are highlighted in black boxes.

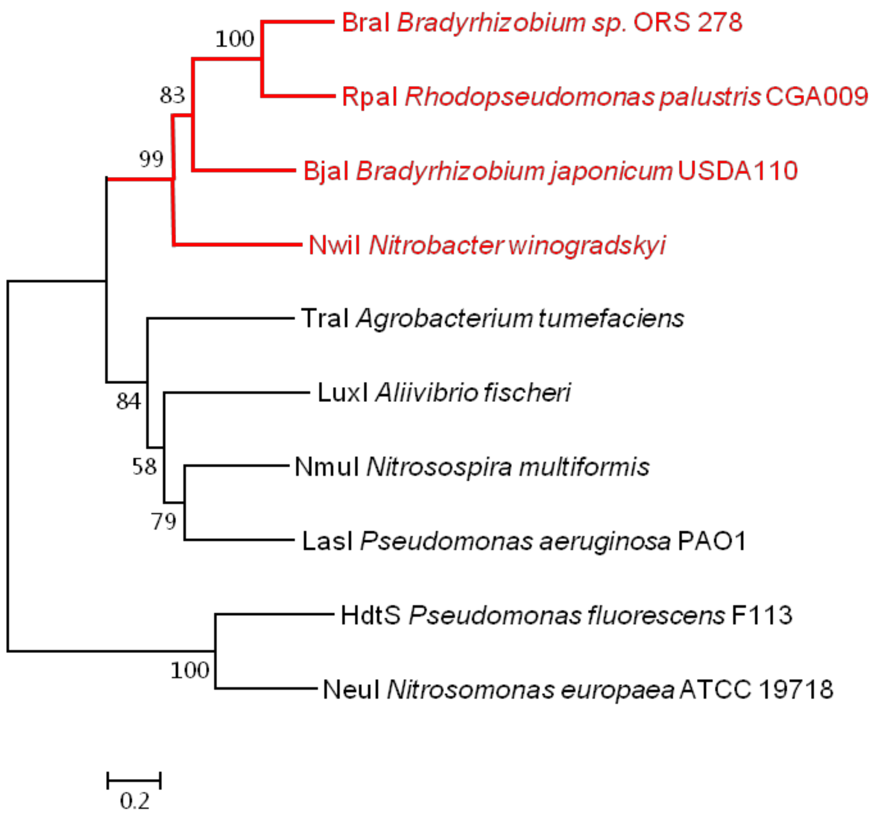

Supplementary Figure S2. Phylogenetic tree of LuxI and HdtS family members. The scale bar indicates the number of substitutions per residue. Bootstrap values as the percentage of 500 samplings are shown for nodes with values of 50% or greater. The subfamily tree of AHL synthases containing BraI, RapI, BjaI and NwiI is highlighted in red.

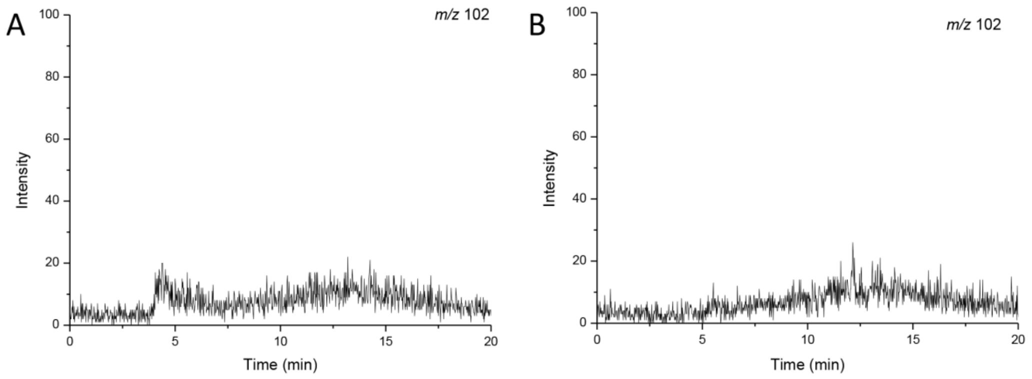

Supplementary Figure S3. LC-MS chromatograms of LB and 756 medium. (A) LB medium. (B) 756 medium.

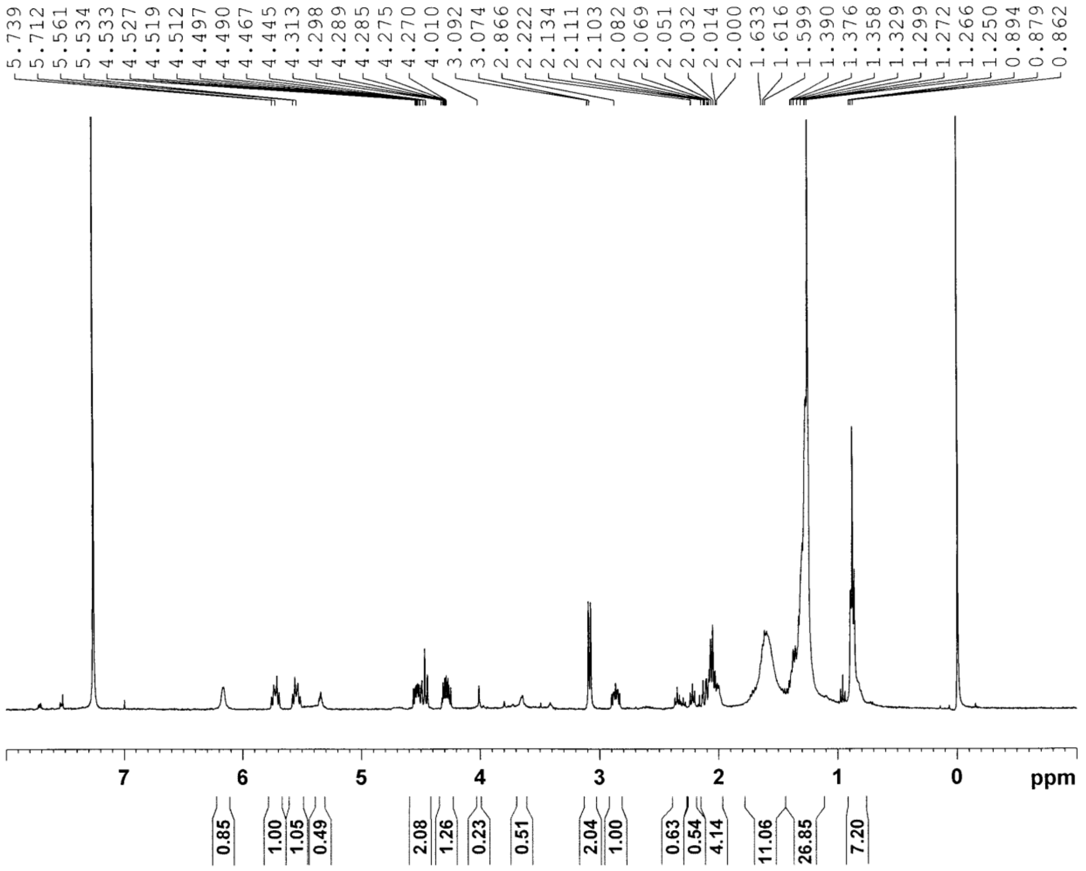

Supplementary Figure S4. ^{1}H NMR purified from the extracts of recombinant *E. coli* strain contain *nwiI* gene. ^{1}H-NMR ($CDCl_3$, 400 MHz) sprctrum: δ_H 0.88(3H, t, a,), 1.29(8H, m, b)， 2.01(2H, m, c), 2.11(1H, m, β), 2.86(1H, m, β), 3.08(2H, d, e), 4.28(1H, m, γ), 4.47(1H, t, α), 4.53(1H, m, γ), 5.54(1H, m, d), 5.73(1H, m, d), 6.17(1H, m, f).

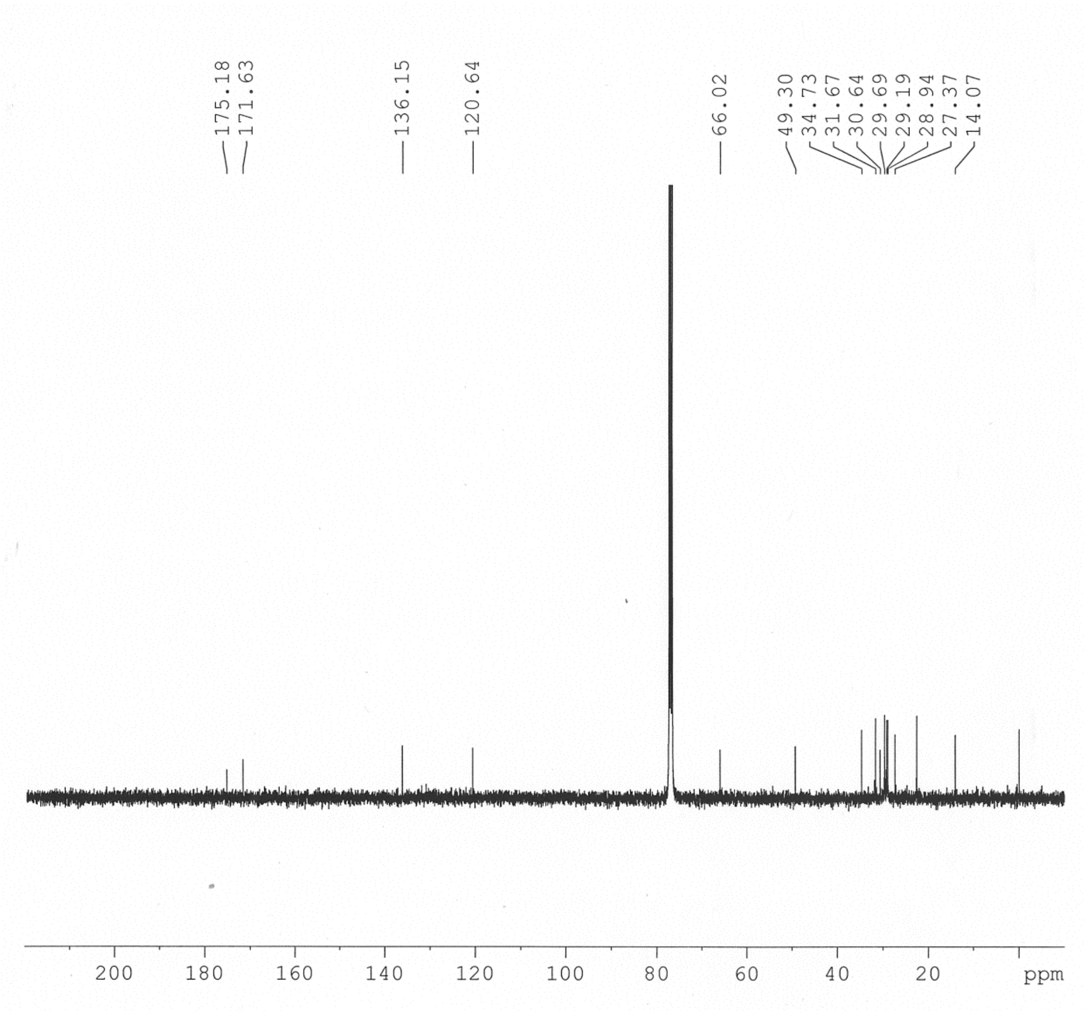

Supplementary Figure S5. ^{13}C NMR purified from the extracts of recombinant *E. coli* strain contain *nwiI* gene. ^{13}C NMR (100MHz, $CDCl_3$): δ 14.07, 27.37, 28.94, 29.19, 29.69, 30.64, 31.67, 34.73, 49.30, 66.02, 120.64, 136.15, 171.63, 175.18.

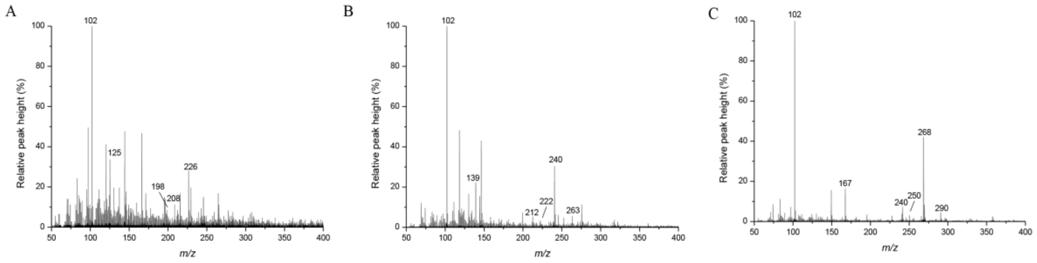

Supplementary Figure S6. LC-MS chromatograms of putative AHLs molecules in *E. coli* containing pGEX-nwiI. (A) C8:1-HSL, (B) C9:1-HSL, (C) C11:1-HSL.

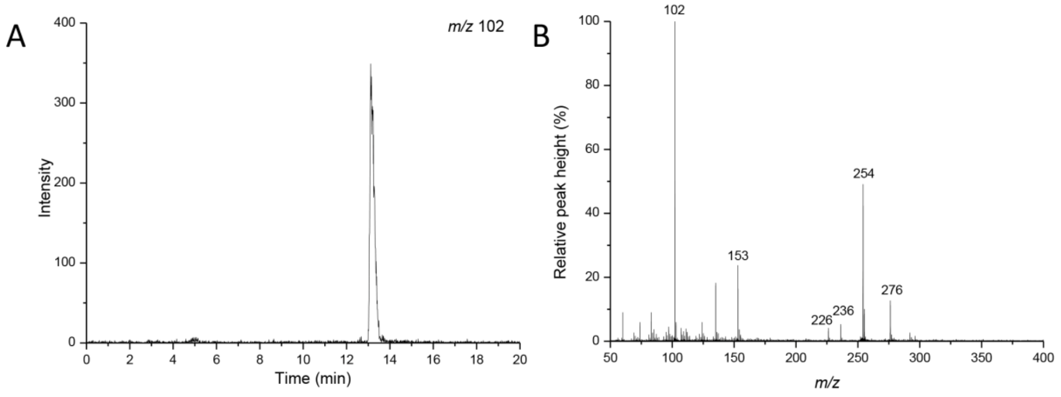

Supplementary Figure S7. LC-MS chromatograms of C10:1-HSL obtained by preparative HPLC. (A) Chromatogram of the lactone moiety at *m/z* 102. (B) The mass spectra reveal molecular ion [M+H] of *m/z* 254. The major fragmentation products with their respective *m/z* are labeled.

Reference

1 Zhu, J., Chai, Y., Zhong, Z., Li, S. & Winans, S. C. *Agrobacterium* bioassay strain for ultrasensitive detection of *N*-acylhomoserine lactone-type quorum-sensing molecules: detection of autoinducers in *Mesorhizobium huakuii*. *Appl. Environ. Microbiol.* **69**, 6949-6953 (2003).
